# IFALD in children: What's new? A narrative review

**DOI:** 10.3389/fnut.2022.928371

**Published:** 2022-07-25

**Authors:** Fabiola Di Dato, Raffaele Iorio, Maria Immacolata Spagnuolo

**Affiliations:** Department of Translational Medical Science, Section of Pediatrics, University of Naples Federico II, Naples, Italy

**Keywords:** intestinal failure, parenteral nutrition, cholestasis, liver transplantation, children

## Abstract

Intestinal failure-associated liver disease (IFALD) is a progressive liver disease complicating intestinal failure (IF). It is a preventable and reversible condition, but at the same time, a potential cause of liver cirrhosis and an indication to combined or non-combined liver and small bowel transplantation. The diagnostic criteria are not yet standardized, so that its prevalence varies widely in the literature. Pathophysiology seems to be multifactorial, related to different aspects of intestinal failure and not only to the long-term parenteral nutrition treatment. The survival rates of children with IF have increased, so that the main problems today are preventing complications and ensuring a good quality of life. IFALD is one of the most important factors that limit long-term survival of patients with IF. For this reason, more and more interest is developing around it and the number of published articles is increasing rapidly. The purpose of this narrative review was to focus on the main aspects of the etiology, pathophysiology, management, prevention, and treatment of IFALD, based on what has been published mainly in the last 10 years. Controversies and current research gaps will be highlighted with the aim to pave the way for new project and high-quality clinical trials.

## Introduction

Intestinal failure-associated liver disease (IFALD) refers to liver injury due to intestinal failure (IF) and parenteral nutrition (PN) occurring in the absence of any other primary parenchymal liver diseases (1). As recently reported by the American Society for Parenteral and Enteral Nutrition (ASPEN), “pediatric IF is the reduction of functional intestinal mass below that which can sustain life, so that the patient depends from supplemental parenteral support for a minimum of 60 days within a 74-consecutive day interval” (2). IFALD is one of the most common and serious complications of IF and consequently of long-term PN (3, 4). The term IFALD replaces the old terminology of “PN-associated liver disease/cholestasis (PNALD, PNAC)”; this condition can be stabilized or reversed with the modifications of nutritional strategies and promotion of intestinal adaptation or it can progress up to end-stage liver disease (2). It is difficult to define the exact prevalence of the disease in childhood due to the limited number of studies, the different criteria used to define IFALD, and the heterogeneity of the populations studied. In the recent years, a better understanding of its pathogenesis has made it possible to know that not only long-term PN, but also IF and its etiology are involved in the development of the disease, even if more is to discover (3). IFALD can present with a wide spectrum of hepatic manifestations, from cholestasis usually in neonates and young children to steatosis and fibrosis in older children and adults. Moreover, it can progress to cirrhosis complicated with portal hypertension and be indication to liver transplantation (4). IFALD is one of the most important determinants of long-term survival in pediatric patients with IF, and for this reason, more and more interest is developing around it. The aim of this narrative review was to focus on the main aspects of the etiology, pathophysiology, management, prevention, and treatment of IFALD in children, based on what has been published mainly in the last 10 years. Controversies and current research gaps will be highlighted with the aim to pave the way for new project and high-quality clinical trials.

## Prevalence, etiology, and risk factors

**Figure 1:**
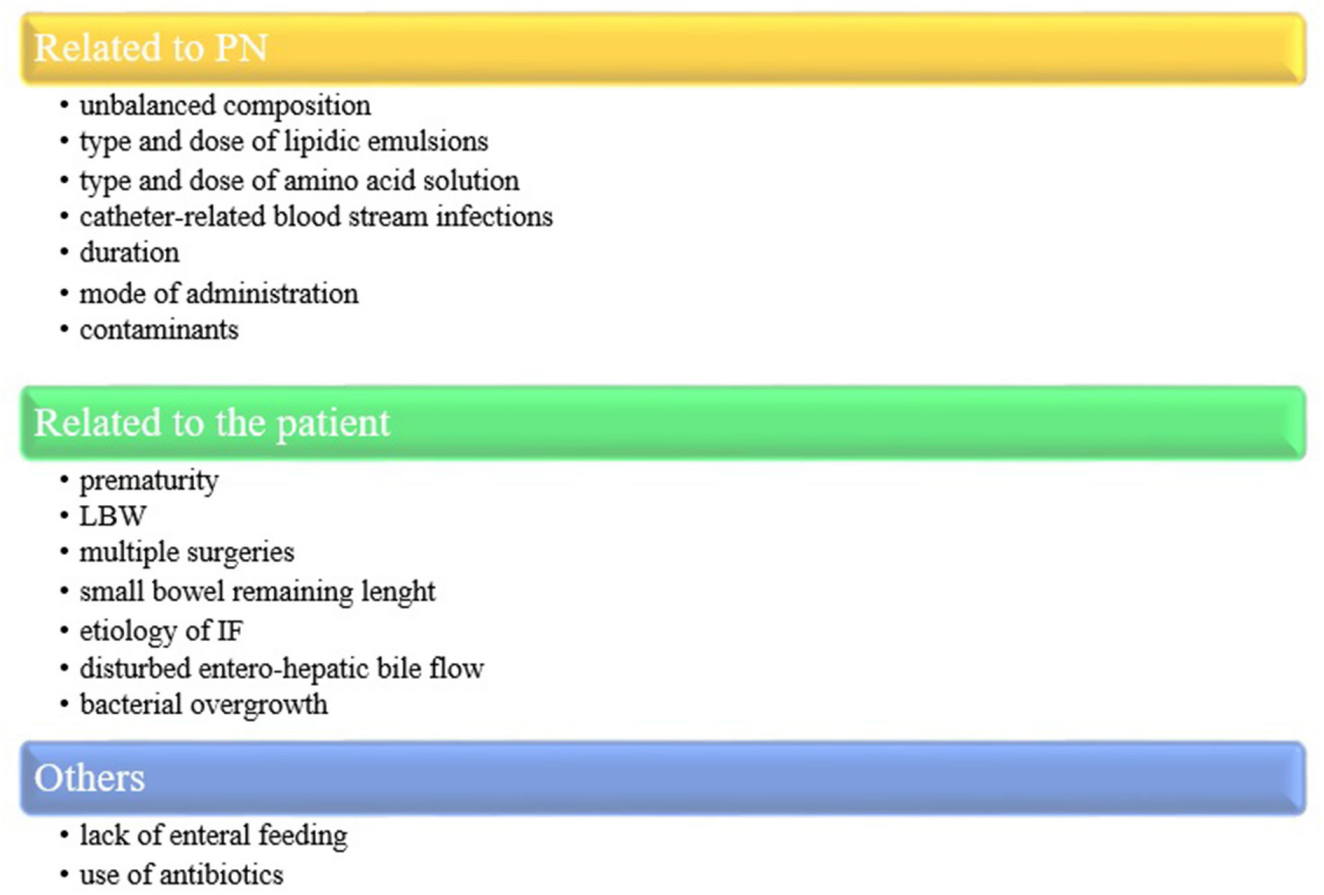
Risk factors for IFALD. PN, parenteral nutrition; LBW, low birth weight; IF, intestinal failure.

The true prevalence of IFALD is difficult to estimate due to the heterogeneity in its definition in different studies (5). It is even more difficult to define IFALD prevalence in the various subgroups of patients according to IF etiology. IFALD occurs in 20%−60% of pediatric patients receiving prolonged PN and manifests as cholestasis in 25%−60% of cases (3, 6–10). In neonatal population, the rates are increased, particularly in patients with necrotizing enterocolitis (NEC). When PN is required for more than 60 days, IFALD occurs in more than 75% of cases (11). Although IFALD has a multifactorial origin, it is widely accepted that soy-based lipid emulsions (SO-LEs), used for parenteral nutrition, play a primary pathogenic role due to their high phytosterol content, high ω-6 to ω-3 long-chain polyunsaturated fatty acid ratio, and low levels of α*-*tocopherol (12). Whereas IFALD was initially considered as a PN complication, an increasing number of evidence suggests that liver disease is not solely due to PN administration but strongly related to IF factors (13). In addition to the components of PN, prematurity, history of multiple surgical procedures, recurrent sepsis, and the lack of enteral feeding are further known risk factors for the development of IFALD (14) (Figure 1).

Intestinal failure results from obstruction, neuromuscular intestinal disorder, surgical resection, congenital defect, or severe protracted diarrhea due to different diseases. In all these cases, the gut is unable to assure the sufficient energetic, macro- and micronutrient, fluid, and electrolyte requirement, and PN represents a life-saving treatment (15, 16). The most common condition of IF necessitating PN is short bowel syndrome (SBS) characterized by significant loss of absorptive surface area usually due to congenital abnormalities such as intestinal atresia, or acquired abnormalities including NEC, volvulus, and mesenteric thrombosis (13).

Neonates, particularly those born preterm, are at greatest risk of IFALD, and it is reported in 28–60% of infants requiring PN for more than 14 days up to 72% when PN is longer than 56 days (15, 17). Prematurity and low birth weight represent the main risk factors for IFALD, with the highest reported incidence in infants born at <34 weeks of gestation and who weigh <2 kg (13). Premature infants who develop NEC are at particular risk for IFALD because, in addition to liver immaturity, they are exposed to multiple risk factors such as higher rate of sepsis, short bowel, disrupted enterohepatic bile acid circulation, enteral feed intolerance, and longer duration of PN (3, 18). Actually, both factors related to IF and PN play a key role in the development of IFALD. Among IF-related factors, NEC with subsequent SBS, multiple surgeries, the lack of enteral feeding, disturbed enterohepatic bile flow, the presence of inflammation, oxidative stress, immaturity of the liver, bacterial overgrowth, catheter-related blood stream infection, and sepsis are the main determinants (3, 5, 15, 17–19). On the other side, imbalance (deficiency/excess) of parenteral nutrients and their composition may contribute to the development of IFALD (5). It is known that type and dose of amino acid solution affect the development of IFALD not only in older children and adults, but especially in neonates. Intravenous lipid emulsions, particularly when administered at high dose and based on soybean or safflower oil, are implicated in IFALD development (3, 20). PN mode of administration and its duration also play a role (14). Although many risk factors are known, the identification of patients at heightened risk for IFALD progression still poses a considerable challenge due to possible evolution to liver cirrhosis of patients with normal liver enzymes and bilirubin values (21). In patients with established IFALD, progression to end-stage liver disease has been described in 4% of patients, and mortality as high as 40% has been reported, representing that severe IFALD is the main indication for intestinal transplant in children (3).

***Desirable future studies****: Prospective observational studies in well-defined setting are desirable to estimate the exact prevalence of IFALD in infants treated with PN as well as older children with long-term PN dependence. National and international networks could also help to define the prevalence of IFALD based on different IF etiologies in long-term PN-treated patients*.

## Pathophysiology

Intestinal failure-associated liver disease pathogenesis is dependent on a variety of host- and PN-related factors (15). Host factors include an immature bile secretory mechanism, bile stasis due to fasting, disruption of the enterohepatic circulation, alterations in the gut microbiome, and repeated septic episodes resulting in endotoxemia (13, 15). Liver immaturity and major susceptibility to damage from lipid peroxidation explain higher risk of IFALD during the first months of life (3). The lack of enteral feeding can be responsible for mucosal atrophy and increased intestinal permeability. The latter can promote the translocation of bacterial lipopolysaccharide promoting inflammation and fibrogenesis (13). Ileum inflammation likely results in dysfunction of farnesoid X receptor (FXR)/fibroblast growth factor (FGF) 19 signaling. FGF19 mediates bile acid homeostasis through a negative feedback on their synthesis, and it is secreted in response to FXR activation which is a BA receptor (13). Xiao et al. (22) showed that children with IF have increased levels of primary BA in blood and liver and lower serum FGF19 concentration compared to controls. Authors suggested that ileum inflammation could cause an impaired intestinal expression of FGF19 and consequent increasing synthesis of BA leading to liver damages. FGF19 correlation with hepatic inflammation and fibrosis was also confirmed by Mutanen et al. (23) in 2015, suggesting its role in IFALD pathogenesis.

The secretion of cholecystokinin can also be reduced, promoting biliary sludging, acalculous cholecystitis, and sometimes bile duct obstruction with consequent liver toxicity (13).

As for PN-related factors, the composition of the used mixtures has a key role in IFALD development. Excess of macronutrients, deficiency of micronutrients, and the different types of lipid emulsions are all important determinants in IFALD pathogenesis (13). Excessive glucose may result in increased plasma insulin, hepatic lipogenesis, fatty liver, and fibrosis, whereas excessive protein may reduce the bile flow (13, 15). Other influencing factors are as follows: excess vitamin A, methionine and manganese, aluminum contamination and deficiencies in taurine, choline, and glutamine (24).

Liver steatosis may also be caused by a deficiency in choline, carnitine, and essential fatty acids (EFAs) (13). EFA deficiency can be related to the amount and different types of lipid emulsions administered. SO-LEs have high concentrations of proinflammatory ω-6 polyunsaturated fatty acids and phytosterols and have been implicated in decreased bile flow and increased bilirubin and serum bile acid levels (25). In fact, the ω-6 polyunsaturated fatty acids lead to Kupffer cell activation and are prone to the oxidation causing liver toxicity (3). Oxidative damage is more evident in SO-LEs even because of their low concentration of the antioxidant α-tocopherol. Phytosterols may produce direct oxidant damage to the liver or may compromise bile acid synthesis and transport probably due to the interference with the FXR (3, 13, 15). Isaac et al. in 2019 (26) demonstrated that lower hepatic phytosterol is hepatoprotective explaining the reduced prevalence of cholestasis observed in piglets receiving fish-oil containing lipid emulsions. Serum and hepatic phytosterol concentrations correlate with cholestasis, inflammation, and fibrosis on liver biopsy in children with IFALD (27). In particular, *in vitro* and *in vivo* studies have identified stigmasterol, present in SO-LE, as playing an important role. Stigmasterol appears to correlate with disease severity and early change in its concentrations predicts later changes in bilirubin values (28).

Fish oil has been considered able to increase anti-inflammatory eicosanoids and reduce triglycerides and inflammation (25). Whether these emulsions allow to avoid or reverse IFALD in children will be discussed in the prevention and treatment section. However, type of lipidic emulsion, molecular alteration of bile acid metabolism, and fecal microbiota seem to play a key role in the development of IFALD, so that in the recent years, many studies have focused on the gut–lipid–liver axis (4). It is assumed that intestinal injury resulting from different conditions induces bacterial overgrowth and abnormal mucosal permeability that leads to increased absorption of proinflammatory substances inducing cholestasis and hepatic fibrosis (29).

***Desirable future studies:*** *Although the number of studies on the gut–liver axis in different liver diseases is constantly increasing, it is desirable a further research effort in the context of IFALD, a peculiar model in which the gut–lipid–liver axis plays a key role, to improve both the understanding of pathogenesis and to identify new therapeutic strategies*.

## Clinical and histological manifestations

Intestinal failure-associated liver disease can manifest with several abnormalities from steatosis, cholestasis, cholelithiasis to fibrosis up to biliary cirrhosis with portal hypertension, liver failure, and rare case of hepatocellular carcinoma (16, 30). At liver histology, infants usually show canalicular cholestasis with biliary obstructive features, ductular reaction, and portal fibrous expansion. Perivenular fibrosis and ductopenia may also be seen in IFALD (13). While the active IFALD may rapidly progress to biliary cirrhosis, the fate of the chronic fibro-steatotic phase is still not precisely known. Progression to end-stage liver disease is uncommon, but not rare in infants and neonates (13, 16). IFALD is a poor prognostic factor for pediatric patients. An increased mortality is reported in patients with IFALD compared to those without it and in children with a more advanced disease. The 3-year survival rate reported is 86% even if Torres et al. have recently reported an overall survival of 97% with 81% of patients achieving enteral autonomy in SBS children on low dose of SO-LEs (31–33).

Usually, cholestasis is prominent in infants and children whereas steatosis and steatohepatitis are more common in older children and adults (13). Not always biochemical enzyme levels correlate with the degree of fibrosis and they can also be normal during disease progression, whereas an association between the severity of the liver injury and the duration of PN is documented (18, 34). In 2021, Mutanen et al. (10) analyzed liver histology in 77 children with IF at a median age of 1.7 years and in 48 of them after almost 3 years. Patients were divided into subjects with active, chronic, or no IFALD, which were present in initial biopsy in 48, 21, and 31% of patients, respectively. In the follow-up biopsy, 52% of patients normalized histology, 23% evolved to a chronic stage, 19% remained stable, and 6.3% progressed to active IFALD (10). In patients with active IFALD, cholestasis and portal inflammation were found in 76 and 70%, respectively; fibrosis or steatosis was observed in 81 and 32% of patients, respectively. The authors observed that older age and PN weaning off predicted chronic IFALD, whereas active IFALD was predicted by PN dependency (10). It is to note that abnormal values of liver enzymes were also found in patients with chronic and no IFALD (10). Other papers showed that in adults, intestinal remnant length, in addition to underlying disease, composition of PN, and patient age, is a risk factor for the development of fibrosis evaluated by FibroScan (35). Other studies were published by Naini et al. and Zambrano et al., and the first one described the histopathologic changes in 89 patients who underwent liver biopsy or transplantation while on PN. It showed perivenular fibrosis as a peculiar hepatic feature of IFALD, while there was no correlation between hepatic biochemical enzymes, duration of PN, and the degree of fibrosis or hepatocellular injury (34). Zambrano et al. (36) analyzed 24 autopsies performed in neonates with history of PN finding a significant correlation between PN duration and liver disease. Finally, Mullick et al. (37) had already suggested a correlation between hepatic histologic findings and duration of PN, even if this relationship remains unclear. Regarding the relationship between the etiology of IF and hepatic histology, Gunnar et al. recently described the development of early steatosis in patients with SBS. While steatosis appears to be more common in older children, it seems to develop in an early phase in infants with SBS, likely related to high glucose and amino acid intakes. Early steatosis may contribute to the development of chronic IFALD and more severe long-term consequences on patients with SBS (38).

Not much data are available about the clinical features of patients with IFALD. In particular, no studies investigated the percentage of patients with pruritus, jaundice, or anicteric cholestasis, and how these symptoms make worse their already poor quality of life.

***Desirable future studies****: Since scant information is available on how many patients with IFALD have signs of portal hypertension and its complications, observational studies on these aspects should be carried out*.

## Diagnosis

There is no consensus on the criteria for diagnosing IFALD. Mild conjugated hyperbilirubinemia may represent the only initial sign and a red flag for disease progression if persistent. Patients with IF without IFALD can also have an isolated mild-to-moderate increase of liver enzymes (3, 39). The presence and severity of the disease is evaluated by combining clinical, biochemical, and imaging findings. Accepted criteria for IFALD include a persistent elevation of transaminases and γ-glutamyl transferase ≥1.5 above the upper limit of normal and hyperbilirubinemia below 3 mg/dl for early IFALD, and persistent hyperbilirubinemia >6 mg/dl and the prolongation of prothrombin time for severe IFALD (39). How long biochemical alterations should persist to define the disease is also debated. Some authors define IFALD as bilirubin > 2 mg/dl after 14 days of PN; others suggest a persistence of biochemical alterations for more than 6 weeks or at least 2–4 weeks (1, 3). Patients with advanced stages of IFALD may have end-stage liver disease complications such as portal hypertension, varices, and hypersplenism. In this case, liver ultrasound can be useful to evaluate spleen size, liver echostructure, and signs of portal hypertension, whereas liver biopsy is not routinely required for diagnosis (1, 3). No clear recommendation is available on the timing and needing of liver biopsy for IFALD diagnosis. Given the lack of consensus diagnostic criteria, it remains a relevant procedure to diagnose IFALD, even if, because of its invasive nature, it is currently limited to individuals with unclear diagnosis or necessity of exact definition of fibrosis stage. Since liver fibrosis is the most important histologic factor conditioning long-term outcome among individuals with different liver diseases, non-invasive markers for the detection of liver fibrosis have developed significantly. Evaluation of non-invasive markers of liver disease in the population at risk for IFALD has the potential to improve our understanding of the development and diagnosis of IFALD. Due to the absence of correlation between histological and biochemical data as reported above, new markers capable of predicting the risk of IFALD progression are desirable. FibroScan (TE) and multiparameter magnetic resonance imaging (MRI) have been studied in adults to assess the severity of steatosis and fibrosis and the correlation with biochemical parameters. TE has been considered a promising method for monitoring the liver injury in adults on long-term PN due to its correlation with biochemical scores (35). Biochemical scores such as aspartate transaminase to platelet ratio index, fibrosis-4 index, gamma-glutamyl transferase to platelet ratio index, and fibrosis index may be useful to assess liver steatosis in adults with IFALD (40).

Recently, Bluthner et al. carried out a prospective study on adults affected by IF to evaluate the ability of non-invasive tests to assess liver function and fibrosis. Patients were grouped in “stable PN” (SPN) and “reduced PN” (RPN) based on their dependence from PN. Due to impaired nutritional status and short remnant bowel length, nine patients were dependent on total parenteral nutrition (SPN) whereas 11 patients were able to halve their parenteral caloric intake over time (RPN). Over time, authors detected a progressive reduction of liver maximum capacity test (LiMax) values in SPN group from baseline to 6, 12, and then 24 months of follow-up (18). LiMax test is a non-invasive diagnostic method for determining enzymatic liver function, based on the ^13^C-labeled metacetin substrate which is metabolized exclusively by cytochrome P450 1A2 to ^13^CO_2_ and acetaminophen (41). Transaminases showed mild reduction in RPN group, while were stable in patients with SPN after 12 months and increased after 24 months. LiMax anticipated the deterioration of the other liver function tests appearing more sensitive in detecting early changes in liver function in comparison with other liver function tests, laboratory-based scores, or imaging-based liver function tests (18). It could be useful to detect early hepatic dysfunction in patients with IF receiving PN. The inefficacy of laboratory-based scores, such as aspartate aminotransferase to platelet ratio index and Fibrosis-4 scores, is due to their correlation with the severity of histological cholestasis more than fibrosis, and the same evidence is reported for the FibroScan, which instead showed encouraging results in children with IFALD (42). However, recently, Micic et al. in their retrospective cohort study found a positive correlation between the FIB-4 index and the liver fibrosis stage as characterized by the Brunt classification. This evaluation of the FIB-4 index against liver biopsies supports the use of the FIB-4 index in the detection of liver fibrosis in IF (43). Few studies have been published on non-invasive tools for assessing liver involvement in IFALD children and the results are reported in Table 1 (10, 42, 44–48).

**Table 1:** Non-invasive tests for diagnosis and monitoring of IFALD in children.

**Author (reference)**	**Patients number**	**Non-invasive** ** test**	**Results**
Mutanen et al. (10)	77	TE/GGT/citrulline	GGT, liver stiffness, and citrulline together had the highest accuracy for detecting active IFALD
Hukkinen et al. (42)	57	APRI/TE	The TE cutoff point was 4.25 kPa for discrimination of any fibrosis and 4.75 kPa for the detection of significant fibrosis. APRI was able to discriminate the presence of histological cholestasis, but was unable to predict any degree of fibrosis
Lawrence et al. (44)	37	Ultrasound Elastography	Positive correlation between stage of fibrosis and mean SWS
Rumbo et al. (45)	36	APRI	APRI score >1.6 predicts advanced fibrosis
Diaz et al. (46)	48	APRI	APRI could significantly predict cirrhosis, but not fibrosis
Hong et al. (47)	63	VCTE	The optimal cutoff to predict moderate/severe liver fibrosis was liver stiffness ≥6 kPa. APRI failed to discriminate mild from moderate to severe fibrosis
Nagelkerke et al. (48)	32	TE/APRI/ ELF	TE measurement correlated positively with age at inclusion, PN duration, weight for age, and AST, while negatively with the amount of infused lipid emulsion. APRI moderately correlated with the number of septic episodes, PN duration, and the percentage of calories delivered *via* enteral nutrition. APRI strongly correlated with AST and ALT and moderately with GGT, total bilirubin, and conjugated bilirubin. ELF score did not correlate with any of the evaluated risk factors. TE measurement moderately correlated with APRI. ELF score did not correlate with TE measurement or APRI

*TE, transient elastography; VCTE, vibration-controlled transient elastography; APRI, aspartate aminotransferase to platelet ratio index; ELF, enhanced liver fibrosis; SWS, shear wave speed*.

***Desirable future studies:*** *Given the invasiveness of liver biopsy and the impact that this procedure can have on patients' quality of life, it is highly desirable to increase studies aimed at validating the use of non-invasive tests for the assessment of liver fibrosis. However, the current trend not to perform liver biopsy commonly compromises an adequate comparison between invasive and non-invasive methods*.

## Prevention and treatment

The main interventions to prevent IFALD are based on early recognition and timely medical management of NEC, aseptic central venous catheter (CVC) care protocols, and early enteral feeding. As for parenteral nutrients, excessive energy intake, dextrose, and amino acids high doses should be avoided because they are related to increased prevalence of IFALD (3).

One of the most important factors to prevent or reverse IFALD is the enteral nutrition and this supports the importance of gut–liver axis in disease pathogenesis. The presence of food in gut promotes mucosal hyperplasia, absorbing surface increase, intestinal motility improvement, and gallbladder contractility. For these reasons, the European Society for Pediatric Gastroenterology Hepatology and Nutrition (ESPGHAN) recommends to start enteral feeding as soon as possible after surgery (3). Earlier introduction of enteral feeding may improve intestinal function reducing IFALD risk and improving overall nutrition status and growth. Shakeel et al. compared 167 infants managed in the period of 2013–2018 in units that followed the guidelines on postoperative feeding and 242 historical controls, observed in the period of 2007–2013. Their study showed that the implementation of guidelines focused on postoperative feeding has a favorable impact on many parameters such as the timing of onset of postoperative enteral feeding, the time to achieve enteral autonomy, the incidence rates of IFALD, the peak of direct bilirubin, the overall use of PN, and the length of hospitalization. Furthermore, faster feeding advancement is associated with significantly decreased IFALD severity, lower peak of direct bilirubin, and higher percentage of IFALD resolution by discharge (11). Breast milk and amino acid-based formula seem to be the best options to re-feed infants with IF. Intermittent feeding is physiological and promotes hormone secretion and cycling gallbladder emptying, but in case of SBS, continuous infusion during the night is suggested with oral bolus to add as soon as possible (3).

Recently, interest in the use of pre- and probiotics for the prevention and treatment of IFALD is increasing, but no randomized clinical trials are available; furthermore, a risk of bacteremia with such supplements has been hypothesized (49). For these reasons, until now, probiotics are not recommended to prevent or treat IFALD (3). Likewise, there is no evidence to support the role of non-absorbable cyclic antibiotic therapy for small intestine bacterial overgrowth to prevent IFALD (29).

On the other hand, avoiding recurrent central line infections has an accepted role in the prevention of IFALD. Parental education and use of taurolidine locks are equally well-accepted prevention strategies (24).

Even the timing of PN infusion plays a role in the prevention of IFALD. Cyclic infusion in patients on long-term PN may reduce the risk of liver disease and reduce liver enzymes and conjugated bilirubin when compared to continuous PN. It has favorable metabolic effects in children younger than 3 years and can revert IFALD, so that nowadays, cyclic PN in stable patients receiving prolonged PN is recommended (3, 50).

### Role of lipid emulsions in IFALD prevention and treatment

Various strategies are used in practice to minimize the incidence of IFALD and development of EFA deficiency, which include reducing lipid dosage, PN cycling, and increasing enteral nutrition in addition to the use of PN (25). The goal of lipid therapy was to ensure adequate nutritional goals by maintaining triglyceride levels <200–400 mg/dl. Many studies and several different formulations have been developed to achieve nutritional aims that minimize the adverse effects.

#### Soybean lipid emulsions (SO-LEs)

It is known that SO-LEs, particularly when administered at high doses, are a risk factor for IFALD. This is due to their high content of ω-6 fatty acids and phytosterols (14). For this reason, its dose has been reduced from ~3 to 1 g/kg/day slowing IFALD progression and ensuring growth and adequate lipid intake. Recently, Gupta et al. carried out a prospective single-center double-blinded randomized controlled trial in infants with gastrointestinal surgical disorders and treated with SO-LE intake of 2 or 1 g/kg/day for 6 weeks. They showed that reducing SO-LE intake is not associated with reduced incidence of IFALD among late-preterm and term infants even if there is a possible risk of earlier development of IFALD with SO-LE intake of 2 g/kg/day. They hypothesized that if PN was continued beyond the study period of 6 weeks in both groups, infants receiving 2 g/kg/day of SO-LEs developed IFALD 1 week earlier than infants receiving 1 g/kg/day of SO-LE intake (51). Baker et al. (20) demonstrated that PN-dependent infants with IFALD receiving low dose of SO-LEs had sufficient EFA without occurrence of EFA deficiency.

#### Mixed oil-based lipid emulsions (MO-LEs)

Later, MO-LEs have been introduced in clinical practice to prevent and treat IFALD. They contain a more balanced ratio of ω-6 to ω-3 fatty acids providing immunomodulatory benefits and might reduce the incidence of cholestasis, hepatic steatosis, and IFALD (14, 18). In 2021, Ferguson et al. (14) evaluated the incidence and severity of IFALD in a highly surgical neonatal population who received MO-LEs and SO-LEs and required long-term PN. A total of 107 patients were included in the study and IFALD occurred in 44.8% of patients receiving SO-LEs compared with 30% of patients receiving MO-LEs. However, the type of lipids was not a significant predictor for the development of IFALD, whereas the duration of PN and duration of lipids administration were the significant risk factors for IFALD, regardless of type of lipid emulsion. No significant differences about the peak of direct bilirubin and hypertriglyceridemia were found (14). The HOME study is a prospective, randomized, controlled, double-blinded, multicenter, international clinical trial ongoing in Europe on adult patients. They will be randomly assigned to receive the n-3 PUFA-enriched medium- or long-chain triglyceride LE or the MCT/LCT LE for a period of 8 weeks. The aim of the study which will end in 2023 was to assess the change in liver function parameters from baseline to final visit and to evaluate the safety and tolerability as well as the efficacy of the LEs (52).

#### Soybean, medium-chain triglycerides, olive oil, and fish oil lipid emulsions (SMOF-LEs)

More recently, the composite SMOF-LE has become available and appears to be well tolerated by infants and children (3). A total of four different lipid sources are present: 30% soybean provides ω-3 and ω-6 for essential fatty acid supply; 30% medium-chain triglycerides have a faster metabolic clearance; 25% olive oil contains monounsaturated fatty acids that are less susceptible to lipid peroxidation; and 15% fish oil supplies the ω-3 long-chain polyunsaturated fatty acids EPA and DHA, which are capable of improving growth, mental and psychomotor development scores, and visual acuity (14).

Daniel et al. found a higher incidence of IFALD with SO-LE compared to SMOF-LE without difference in the time of development. Moreover, in the IFALD group of patients, they showed higher AST and bilirubin values in patients with SO-LE without other difference in liver function tests. This study showed that there was a 20% higher incidence of IFALD for patients receiving SO-LE than for patients who received SMOF-LE. It is to note that patients with SO-LE appeared to be younger with a higher risk of developing IFALD; moreover, the role of different etiologies and the small sample size could have influenced the high rates of IFALD in the SO-LE group (25). A large prospective randomized study showed lower bilirubin values in infants treated with SMOF-LE compared to patients on SO-LEs, demonstrating protective effects of SMOF-LE on the liver (53). Other studies have compared SMOF-LEs and SO-LEs for safety, efficacy, and tolerability in premature neonates, infants, and children with discordant results showing on the one side no difference in serious adverse events, lipid profile, growth parameters, and total bilirubin and on the other side beneficial effects of SMOF-LEs on cholestasis prevention. A meta-analysis showed that there was no difference in the rate of cholestasis or bilirubin levels with short-term use between different LEs, but the use of SMOF-LEs might be beneficial when PN is expected to be longer than 2 weeks (54). However, guidelines advice against the SO-LEs use in preterm infants, newborns, and older children on short-term PN because of less balanced nutrition, suggesting composite LEs with or without fish oil in case of PN lasting longer than a few days (19).

#### Fish oil lipid emulsions (FO-LEs)

It has been reported that fish oil is able to improve biliary flow in animal models and reduce *de novo* lipogenesis, stimulate beta-oxidation, and decrease hepatic steatosis (3). In fish oil, there is a high concentration of α-tocopherol and the absence of phytosterols; it contains low amount of EFA and high dose of ω-3 fatty acids (3). It should be noted that the composition of these emulsions raises the concerns about the deficiency of EFA whose risk is greater in infants with IF as a consequence of their malabsorption and if an adequate parenteral supply of EFA is not offered. For these reasons, especially for long-term treatment, mixed LEs are preferred giving adequate linoleic acid and α-linolenic acid and a more moderate balance of ω-3:ω-6 fatty acids (17).

Two systematic reviews were produced in 2019 to compare the safety and efficacy of all LEs for PN in all preterm and term infants. In preterm infants, meta-analysis found no difference in the incidence of PNALD/cholestasis between FO-LEs and all non-FO-LEs. In preterm infants with PNALD/cholestasis, the meta-analysis showed significantly less cholestasis with the use of FO-LE compared to SO-LE. However, these were studies with a small number of patients and with methodological differences. In conclusion, there is no particular advantage using LEs with or without fish oil for the prevention of PNALD/cholestasis, growth, mortality, retinopathy of prematurity, bronchopulmonary dysplasia, and other neonatal outcomes in preterm infants (55). In term and late preterm infants with surgical condition, no difference was found in incidence of PNALD/cholestasis, whereas in infants with PNALD, FO-LEs were associated with better weight gain (56). In both the papers, there were no differences in death or sepsis between FO-LEs and SO-LEs with very low-quality evidence grade (55, 56).

Considering the importance of lipids for infant growth and development, several studies evaluated the impact of lipid restriction or different formulations on somatic growth in infants and children. Raphael et al. in 2020 (57) compared the natural history of growth in infants treated with FO-LE with published growth standards as well as with a historical cohort of infants treated with SO-LE. They observed that FO-LE subjects were more likely to achieve enteral autonomy and less likely to experience liver/bowel transplantation or death compared with the SO-LE group. Furthermore, suboptimal growth patterns were registered until 11 months postmenstrual age in both FO-LE and SO-LE groups; the FO-LE group showed a greater catchup gain in weight, length, and head circumference, so that an adequate somatic growth can be obtained with FO-LE treatment (57).

As reported above, children with IF have also higher risk of gallstones. Mixed LEs are also associated with a lower incidence of biliary sludge and gallstones in comparison with children on SO-LEs (58).

### Role of vitamin K supplementation

Children with IFALD are at risk for vitamin K deficiency during cholestasis. In particular, during reversal phase, close monitoring and quantified supplementation of vitamin K may be warranted (59). However, Ghirardello et al. in 2020 (60) showed that preterm newborns with IFALD do not show coagulation impairment when receiving oral vitamin K supplementation by fortified milk or formula.

### Treatment of IFALD

A retrospective analysis of 31 children with irreversible IF referred for intestinal transplantation showed that IFALD improvement was achieved with the changes in patient management such as reduction in SO intake, implementation of cyclical PN infusion, addition of α-tocopherol to PN solutions, managing of bacterial overgrowth, and promotion of enteral nutrition (61).

Currently, ESPEN/ESPGHAN/ESPR/CSPEN guidelines on pediatric PN suggest to suspend SO-LEs or reduce other LE doses or use composite LEs containing fish oil to treat children with IFALD (19). Even if an increasing number of evidence is available about the possible reversion of cholestasis after discontinuation of PN or following changes in lipid management, it is to note that Mercer et al. in 2013 (62) showed that the reversal of hyperbilirubinemia with FO-LEs does not reflect a similar histologic regression of fibrosis. Portal fibrosis and steatosis persist in 45 and 55% of the patients, respectively, as showed by Mutanen et al. (63). Several studies indicate that reduction in SO-LE to ≤ 1 g/kg/day can reverse IFALD, and the reversion in 3–6 months has been documented in 75% children after switching to FO-LE at 1 g/kg/day when compared to SO-LE at 2–3 g/kg/day (64, 65). Pure FO-LEs are considered a treatment that can reverse IFALD, increase survival, and avoid the need for transplantation, so that they are considered a rescue therapy (17, 19, 66). In particular, mixed LEs with fish oil are considered the first-line treatment in patients with cholestasis, whereas the switch to the pure FO-LEs should be considered as rescue therapy in case of persistent hyperbilirubinemia (66). In 2021, a retrospective study on predictors of cholestasis or IFALD response to SMOF-LE therapy in patients with IFALD showed that 38% of the patients treated with SMOF had cholestasis resolution, 7% improvement, and 45% no response. The responders were older at the beginning of SMOF use, treated with it for longer time, with higher corrected gestational age and lower direct bilirubin levels at the start of treatment (12). Previous studies had already demonstrated that younger age, increased direct bilirubin, and signs of liver disease were associated with an increased risk of therapy failure. This work suggested that early initiation of SMOF therapy, when cholestasis is less severe, improves the likelihood of resolution (12). Regarding use of FO-LEs for IFALD treatment, Nandivada et al. demonstrated that most PN-dependent infants with IFALD respond to FO therapy with the resolution of biochemical cholestasis and avoidance of liver transplantation. In their study, 86% achieved resolution of cholestasis and 14% failed therapy; the latter were older, with median lower birth weight and advanced liver disease. They stated that a PELD score >15, history of gastrointestinal bleeding, age at FO initiation >16 weeks, the presence of non-gastrointestinal comorbidities, and mechanical ventilation at FO initiation were the independent predictors of treatment failure (67).

#### Pharmacological Treatment

Between the commercially available drugs, oral erythromycin showed to reduce the incidence of IFALD in premature infants due to its effect on gastrointestinal motility and intestinal microbiome. Obeticholic acid, a semisynthetic bile acid, has been proposed as IFALD treatment for its role as an FXR agonist (29). Another oral bile acid, ursodeoxycholic acid (UDCA), has been used in the prevention and treatment of IFALD since the early 90s. UDCA is a bile acid which appears to have a direct cytoprotective effect and immunomodulatory properties and to reduce the hydrophobic bile acids in the hepatobiliary system to lessen the potential for hepatotoxicity. However, there is still less data to recommend its prophylactic use to prevent IFALD (68). As for IFALD treatment, UDCA showed to improve liver function tests first in adults (69). Later, its safety and efficacy in decreasing bilirubin and liver enzymes values were also confirmed in children and then in premature newborns undergoing prolonged PN and very-low-birth-weight infants (70–72). The efficacy of the drug has been questioned because of intestinal ability to absorb it in patients with IF (68). In 2020, Mouillot et al. addressed the topic again. They carried on a pilot study in five patients with SBS with normal liver function tests showing how, in these patients, UDCA decreases ALT values and the hepatic synthesis of triglycerides and cholesterol with consequent prevention of IFALD onset (73). In conclusion, UDCA may improve the biochemical and clinical symptoms of IFALD even if optimal dosing, duration of therapy, and long-term outcomes require further studies to recommend its routinary use.

#### New drugs for IFALD

Several new molecules are under investigation to assess their possible role for the treatment of IFALD. GW4064 has showed to prevent PNAC in mice through the restoration of hepatic FXR signaling, supporting the hypothesis that augmenting FXR activity may be a therapeutic strategy to alleviate or prevent PNAC. When added to PN, it prevented hepatic injury and cholestasis and normalized serum bile acids. Treated mice also showed significantly reduced serum AST, ALT, total serum bile acids, and bilirubin (74). Other potential therapeutic approaches are the administration of GLP-2 analogs which stimulate intestinal adaption and absorption and thereby lead to reduced reliance on parenteral support and cholestasis (75, 76). A phase III study evaluated the safety and efficacy of teduglutide, a recombinant human GLP-2 analog, in pediatric patients with short bowel syndrome-associated intestinal failure (SBS-IF). The trial included two randomized, double-blinded teduglutide dose groups and a non-blinded standard of care (SOC) group. The safety profile of teduglutide was similar to that reported previously in children and adults. Treatment with teduglutide was associated with significant reductions in parenteral support for pediatric patients with SBS-IF over 24 weeks. Significantly more patients with SBS-IF treated with teduglutide than those on SOC achieved the primary end point of a 20% reduction in parenteral support volume at week 24 (75). Bioletto et al. (77) in their meta-analysis showed a response rate of 64% at 6 months, 77% at 1 year, and 82% at ≥2 years in adult patients with SBS whereas the weaning rate was estimated as 11% at 6 months, 17% at 1 year, and 21% at ≥2 years. Intestinal anatomy was identified as a significant predictor of outcomes. The pediatric network for IF of the Italian Society for Pediatric Gastroenterology Hepatology and Nutrition has provided a position paper on the use of Teduglutide in pediatric IF with the main aim to identify the best candidates from a cost-effective perspective. It has been hypothesized that in children, as compared to adult patients, the changes determined by teduglutide could persist after discontinuation due to continuous growth processes of the intestine (78).

Hepatocyte growth factor effect was studied in a rat model of PNALD. It showed to reduce lipid droplets at histological evaluation of the liver when administered intravenous at low and high dosage in rats (79). IFALD may also benefit from free fatty acid receptor-targeted therapies; ω-3 fatty acids are highly concentrated in fish, seafood, nuts, and plant oils and have been used successfully in treating liver disease contributing to the reduction in liver transplantation and mortality for pediatric patients with IFALD (80).

#### Surgery treatment

Surgery should be considered with the caution especially in children with advanced IFALD because of the high risk of bleeding in case of portal hypertension. The main therapeutic options are restoration of bowel continuity and isolated or combined liver and/or gut transplantation. Liver transplantation seems to be effective in improving gut function, and bowel operations may have effects on liver disease especially if they allow the switch from PN to enteral nutrition improving enteral tolerance, bowel stasis, and the enterohepatic circulation (24). Before surgery, the medical team should consider that the greatest growing potential of the gut is in the first year of life and the optimal timing of lengthening procedure is still unknown. Data to support non-transplant surgery for preventing or improving IFALD are few (3). In 2020, a review by Capriati et al. assessed the efficacy of reconstructive surgery on weaning off PN, survival, development of IFALD, and need for transplantation. As for IFALD, the authors found development of liver disease extremely variable between 2.7 and 67%. Concerning the outcome of surgery, IFALD appeared to be more frequent in the group treated with PN alone than in the groups receiving PN associated with any surgery (5).

Guidelines suggest recurring to bowel reconstruction when there is limited enteral feed tolerance, progressive IFALD, and recurrent sepsis. About isolated small bowel transplant, it should be considered in the absence of severe IFALD when combined transplantation is considered. However, the requirement for significant immunosuppression and the risk of graft rejection still make the transplant a challenging procedure even if the patients' survival has increased (81). The isolated small bowel transplant still represents a challenging procedure, and it is considered less and less. The main reason is that children with intestinal failure have 85% chance of survival on PN and those with SBS who survive the first 3 years of life have a >75% chance of weaning off PN. In fact, recently, it has been reported that 64% of patients with SBS successfully weaned from PN after a median period of 11 months and the percentages increase at 78 and 93% within 3 and 5 years, respectively. In the same work, the survival rate was 84% over 20 years in patients with chronic IF managed with long-term PN with reduced mortality related to the reduction in the volume of lipid administered and the use of FO-LEs. The authors found that IFALD was the main predictor of poor outcome, so that patients with severe and refractory IFALD represent the group in which intestinal transplantation may offer benefit (82). There is evidence about liver fibrosis regression after intestinal transplantation, probably due to the intestinal permeability and gut–liver axis improvement (29). Regarding isolated liver transplantation, some criteria were established to predict its success: established severe IFALD, at least 50 cm of functional small bowel without ileocecal valve or 30 cm with the valve, at least 50% of estimated daily intake tolerated as enteral nutrition and weight gain, and the absence of recurrent line infections (3). The outcome of isolated liver transplantation in patients with intestinal failure and IFALD is better than the outcome of patients who undergo either isolated intestinal transplantation or combined liver and small bowel transplantation. Furthermore, isolated liver transplantation may improve intestinal adaptation reducing the need for small bowel transplantation (83). Because of the good survival rate and overall results in children with IF due to SBS managed with home PN, isolated liver transplantation should not become the SOC, should be considered with extreme caution, and proposed when liver disease with portal hypertension is considered the major obstacle to the enteral autonomy (3, 84, 85). In fact, Taha et al. in 2012 (84) reported the outcome of isolated liver transplantation in eight survivors of 14 liver transplanted children for IFALD. The overall 5-year survival in the whole cohort was 57%. In this study, only one-third of the cohort could be weaned from PN and only three of the five survivors switched from EN to full oral feeding. Authors underline the necessity for these patients of intermittent PN, close follow-up, and the risk of complications. Also, the catch-up growth of the children was no satisfactory in the long term (84). Moreover, the use of FO-LEs has also been associated with lower short-term mortality and decreased need for organ transplantation (86). However, in the rare circumstances that it needs to be exercised as an option, liver transplant should be performed in experienced intestinal failure/rehabilitation centers with close links to intestinal transplant teams (84). Combined liver and intestine or multivisceral transplantation is considered in case of complete intestinal aganglionosis, intestinal pseudo-obstruction, or severe portal hypertension. Unfortunately, intestinal transplantation outcomes are not optimal. In a study by Norsa et al., survival in pediatric patients with SBS was 78%, with mortality occurring only following intestinal transplant, as compared to 100% survival in those on long-term home PN with no life-threatening complications requiring transplantation. The study confirms that long-term PN is the first-line treatment for SBS children with good long-term prognosis compared to intestinal transplantation. A multidisciplinary intestinal rehabilitation team composed by gastroenterologists, dieticians, pharmacists, PN-specialized nurses, and both transplant and non-transplant surgeons may be beneficial in the management of these children (87).

***Desirable future studies:*** Much has been done but much remains to be done in the field of IFALD treatment. If teduglutide appears to be a new and effective therapeutic tool for these patients, long-term data are lacking. Multicenter randomized studies are also desirable to evaluate the role of UDCA in the prevention and treatment of IFALD. Finally, it would be desirable to have a greater quantity of published data relating to the indications and outcome for intestinal, hepatic, or combined transplantation of patients with IFALD, evaluating any likely prognostic factor in this regard.

## Conclusion

In the recent years, more attention has been paid to the outcome and management of IFALD in children. There is still controversy over various aspects of IFALD, including its definition. Well-defined and widely accepted diagnostic criteria are the key to obtaining more accurate data on disease prevalence and complications and to planning reproducible and comparable studies. Pending high-quality clinical studies aimed to clarify the efficacy of different short- and long-term therapeutic strategies, appropriate management of lipid mixtures, early initiation of enteral nutrition, reduction of catheter-induced sepsis, and parenteral cycling programs are improving our ability to prevent and manage this potentially serious complication of bowel failure and are enabling us to move patients as far away from the bowel transplant spectrum as possible.

## Author contributions

All authors conceptualized this narrative review, analyzed current literature, drafted the initial manuscript and revised the final version.

## Conflict of interest

The authors declare that the research was conducted in the absence of any commercial or financial relationships that could be construed as a potential conflict of interest.

## Publisher's note

All claims expressed in this article are solely those of the authors and do not necessarily represent those of their affiliated organizations, or those of the publisher, the editors and the reviewers. Any product that may be evaluated in this article, or claim that may be made by its manufacturer, is not guaranteed or endorsed by the publisher.
